# Kidney microRNA Expression Pattern in Type 2 Diabetic Nephropathy in BTBR Ob/Ob Mice

**DOI:** 10.3389/fphar.2022.778776

**Published:** 2022-03-16

**Authors:** Lucas Opazo-Ríos, Antonio Tejera-Muñoz, Manuel Soto Catalan, Vanessa Marchant, Carolina Lavoz, Sebastián Mas Fontao, Juan Antonio Moreno, Marta Fierro Fernandez, Ricardo Ramos, Beatriz Suarez-Alvarez, Carlos López-Larrea, Marta Ruiz-Ortega, Jesús Egido, Raúl R. Rodrigues-Díez

**Affiliations:** ^1^ Renal, Vascular and Diabetes Research Laboratory, IIS-Fundación Jiménez Díaz, Spanish Biomedical Research Centre in Diabetes and Associated Metabolic Disorders (CIBERDEM), Universidad Autónoma de Madrid, Madrid, Spain; ^2^ Facultad de Ciencias de la Salud, Universidad de Las Américas, Concepción, Chile; ^3^ Molecular and Cellular Biology in Renal and Vascular Pathology, IIS-Fundación Jiménez Díaz-Universidad Autónoma Madrid, Madrid, Spain; ^4^ Laboratorio de Nefrología, Facultad de Medicina, Universidad Austral de Chile, Valdivia, Chile; ^5^ Department of Cell Biology, Physiology and Immunology, University of Cordoba, Maimónides Biomedical Research Institute of Cordoba (IMIBIC), UGC Nephrology, Hospital Universitario Reina Sofía, Córdoba, Spain; ^6^ Centro de Biología Molecular “Severo Ochoa” (CSIC-UAM), Viral Vectors Service, Madrid, Spain; ^7^ Unidad de Genómica Fundación Parque Científico de Madrid, Universidad Autónoma de Madrid, Madrid, Spain; ^8^ Translational Immunology Laboratory, Health Research Institute of Asturias (ISPA), Oviedo, Spain; ^9^ Department of Immunology, Hospital Universitario Central De Asturias, Oviedo, Spain

**Keywords:** miRNA, inflammation, diabetes, type 2 diabetes, diabetic nephropaty, chronic kidney disease, BTBR ob/ob mice

## Abstract

Diabetic nephropathy (DN) is the main leading cause of chronic kidney disease worldwide. Although remarkable therapeutic advances have been made during the last few years, there still exists a high residual risk of disease progression to end-stage renal failure. To further understand the pathogenesis of tissue injury in this disease, by means of the Next-Generation Sequencing, we have studied the microRNA (miRNA) differential expression pattern in kidneys of Black and Tan Brachyury (BTBR) ob/ob (leptin deficiency mutation) mouse. This experimental model of type 2 diabetes and obesity recapitulates the key histopathological features described in advanced human DN and therefore can provide potential useful translational information. The miRNA-seq analysis, performed in the renal cortex of 22-week-old BTBR ob/ob mice, pointed out a set of 99 miRNAs significantly increased compared to non-diabetic, non-obese control mice of the same age, whereas no miRNAs were significantly decreased. Among them, miR-802, miR-34a, miR-132, miR-101a, and mir-379 were the most upregulated ones in diabetic kidneys. The *in silico* prediction of potential targets for the 99 miRNAs highlighted inflammatory and immune processes, as the most relevant pathways, emphasizing the importance of inflammation in the pathogenesis of kidney damage associated to diabetes. Other identified top canonical pathways were adipogenesis (related with ectopic fatty accumulation), necroptosis (an inflammatory and regulated form of cell death), and epithelial-to-mesenchymal transition, the latter supporting the importance of tubular cell phenotype changes in the pathogenesis of DN. These findings could facilitate a better understanding of this complex disease and potentially open new avenues for the design of novel therapeutic approaches to DN.

## Introduction

Diabetic nephropathy (DN) represents the leading cause of chronic kidney disease (CKD), being the most frequent origin of end-stage renal disease (ESRD) and the main reason for renal replacement therapy in Western countries (Cheng et al., 2021; Deng et al., 2021). Nowadays, the incidence of DN continues to grow due to the increasing prevalence of type 2 diabetes (T2D) linked to metabolic syndrome, obesity, and dyslipidemia (Navarro-González et al., 2011; Alicic et al., 2017; Sharma et al., 2017). At present, the main therapeutic targets comprise blood pressure and hyperglycemia control, including the inhibition of the renin-angiotensin-aldosterone system (RAAS) as a nephroprotective strategy (Ruiz-Ortega et al., 2020). During the last few years, sodium-glucose cotransporter-2 inhibitors (SGLT2i) or glucagon-like peptide-1 receptor agonists have been used as the first and second therapeutic options in specific CKD stages. They have shown noticeable beneficial effects on cardiovascular and renal outcomes with a significant decrease in mortality (Neumiller et al., 2017). Apart from these mentioned drugs, the dipeptidyl peptidase 4 inhibitors are also a therapeutic approach currently recommended in the new KDIGO guidelines (Kidney Disease: Improving Global Outcomes Diabetes Work Group, 2020). In addition, new anti-inflammatory therapies and metabolic modulators, such as JAK/STAT, Rho-kinase and Sirtuin-3 inhibitors, peptide N-Acetyl-Seryl-Aspartyl-Proline, glycolysis inhibitors, and mineralocorticoid receptor antagonists, have been postulated as potential drugs in DN due to the preclinical evidence, showing their renoprotective role or even their ability to reverse established renal damage (Hashimoto et al., 2010; Bakris et al., 2020; Locatelli et al., 2020; Opazo-Ríos et al., 2020b; Srivastava et al., 2020; Matoba et al., 2021). Growing evidence in all these mentioned studies has shown that the reno-protective effects of these drugs, at least, in part, may be due to the regulation of epithelial and endothelial-to-mesenchymal transition (EMT and EndoMT) in the progression of DN. Unfortunately, at present, there are no interventions capable of fully prevent the progression to advanced kidney disease in people with T2D, being therefore necessary to find out new therapeutic options that limit the natural history of the disease.

Many studies have investigated the molecular mechanism involved in the onset and progression of DN. Hyperglycemia has been considered the driving and triggering force in the DN development, but recent data have also pointed out an important role of the immune system and inflammation, as well as oxidative stress, lipotoxicity, and uremic toxins (Moreno et al., 2018; Opazo-Ríos et al., 2020a; Lavoz et al., 2020b; Rayego-Mateos et al., 2020). At the molecular level, the importance of signaling pathways related to renal inflammation and fibrosis such as the loss of endothelial glucocorticoid receptor (Srivastava et al., 2021b), endothelial FGFR1 and SIRT3 signaling (Li J. et al., 2017; Srivastava et al., 2018), TGFβ/SMAD, Notch, WNT/β-Catenin, and Sonic-Hedgehog pathways (Marquez-Exposito et al., 2018; Zhao et al., 2018; Wang et al., 2022) have also demonstrated to play a relevant role in DN. Interestingly, despite good glycemic control, the inability to fully avoid chronic meta-inflammation (microinflammatory milieu caused by metabolic factors) could contribute to ESRD progression in diabetic patients. This phenomenon can be explained by the concept of *hyperglycemic “metabolic memory”* (Bheda, 2020). Recent evidence suggests epigenetic regulation mechanisms, including DNA methylation and the histone post-translational modifications, as drivers of metabolic memory, suggesting that epigenetic regulation may result very relevant in DN (Keating and El-Osta, 2013; Zhao et al., 2016; Morgado-Pascual et al., 2018; Martinez-Moreno et al., 2020a; Martinez-Moreno et al., 2020b). Epigenetic regulation of gene expression is a dynamic process that may be modified in response to the environment or therapeutic modulation. In particular, lysine histone methylation, acetylation, and crotonylation have been involved in kidney diseases (Martinez-Moreno et al., 2020a). In addition, epigenetic readers that identify and interpret epigenetic signals are key components of the system (Morgado-Pascual et al., 2019).

Some authors also include microRNAs (miRNAs) in the epigenetic regulation of gene expression. miRNAs are evolutionarily conserved small (20–24 nt) non-coding RNAs related to both stability and translation of target mRNAs. Since their discovery, miRNAs have progressively turned out to central stage in the understanding of the post-transcriptional gene expression regulation. Interestingly, epigenetic regulation modulates several miRNAs expression, and, conversely, some of them participate in the expression of relevant epigenetic regulators (Sato et al., 2011). The importance of miRNAs function has been demonstrated in a wide variety of processes including, cancer, cardiovascular and renal diseases, peritoneal fibrosis, and diabetes (Adams et al., 2014; Morishita et al., 2016; Prattichizzo et al., 2016; Colpaert and Calore, 2019; Ruiz-Ortega et al., 2020; Jankauskas et al., 2021). Regarding DN, some authors have suggested a potential role of miRNAs in the development and progression of the pathology, featuring some of them (miR-21, miR-200b/c, and mir-29c) (Long et al., 2011; Park et al., 2013; Zhong et al., 2013) as promising therapeutic targets, but some others (e.g., miR-146a) (Bhatt et al., 2016a) as protectors of DN progression and fibrosis. In this sense, several miRNAs have been found to modulate EMT and EndoMT and, in consequence, to prevent fibrosis in DN and other kidney diseases by targeting several genes related to these processes (Srivastava et al., 2013; Giordo et al., 2021). Thus, miR-30a, miR-30c, miR-26a, miR-130, miR-23b, Let-7days, and miR-98 have shown to modulate EMT in experimental DN (Bai et al., 2016; Liu et al., 2016; Zheng et al., 2016; Zhu et al., 2019; Gao et al., 2020; Wang Y. et al., 2021). Moreover, the anti-fibrotic effects of other miRNAs, including miR-200a, miR-455-3p, miR-92days-3p, miR-130b, miR-26a, and miR-29a, have been also described in DN (Du et al., 2010; Wang et al., 2011; Lin et al., 2014; Koga et al., 2015; Bai et al., 2016; Wu et al., 2018; Zhang, 2021). Apart from miRNAs, other non-coding RNAs have been postulated to regulate gene expression in DN. In this way, long-noncoding RNAs (lncRNAs) and circular RNAs have been described to act as miRNA sponges, regulating their actions in DN (Srivastava et al., 2021a; Patil et al., 2021). For instance, lncRNAs NR_033515, NEAT1, OIP5-AS1, and MALAT1 promote EMT and fibrosis by sponging anti-fibrotic miRNAs (Gao et al., 2018; Liu et al., 2019; Wang et al., 2019; Li N. et al., 2020; Fu et al., 2020; Meng et al., 2020), whereas other lncRNAs, such as ZEB1-AS1, prevent EMT and fibrosis in DN (Meng et al., 2020). In consequence, different non-coding RNAs and other epigenetic mechanisms can interact to regulate gene expression and, therefore, DN progression. These data, as well as their higher stability and the possibility of measuring them in different fluids, have pointed out their utility as biomarkers for early detection and progression of DN, suggesting their potential use as therapeutic agents (Simpson et al., 2016).

However, an important handicap to be dodged in the study of DN and its complications is the lack of robust animal models that replicate the key features of human diabetes to test novel therapeutic tools (Brosius and Alpers, 2013). In this regard, Black and Tan BRachyury (BTBR) ob/ob (leptin deficiency mutation) mouse has recently turned out as an excellent preclinical model for DN study, because it replicates the key histopathological features observed inT2D (Hudkins et al., 2010; Alpers and Hudkins, 2011), despite the absence of tubulointerstitial fibrosis observed in advanced human DN (PMID: 24711709). Therefore, to further advance in the better knowledge on the mechanisms involved in the genesis and progression of DN, the aim of this study was to characterize by the Next-Generation Sequencing (NGS) the miRNA differential expression pattern in an established model of advanced DN in the BTBR ob/ob mice.

## Materials and Methods

### Ethics Statement

All animal procedures were performed according to the guidelines of animal research in the European Community and with prior approval by the Ethics Committee of the Health Research of the IIS-Fundación Jiménez Díaz and by the Madrid regional government (Ref. PROEX 079/18). All animal procedures conformed to EU Directive 2010/63EU and the national rule 53/2013 regarding protection of animals used for experimental and other scientific purposes. The establishment and care of BTBR ob/ob diabetic and obese mice colony (referred to here as “diabetic mice”) and their corresponding controls [BTBR wild type (WT)] have been previously described (Clee et al., 2005). These mice were originally obtained from JAX™ Mice (Charles River Europe laboratory), and then, the mouse colony breeding was maintained in the Fundación Jiménez Díaz Animal facilities, following the JAX™ recommendations (Jackson Laboratory, 2007). Animals were housed at a density of four animals per cage in a temperature-controlled room (20°C–22°C) with 12-h light–dark cycles and feeding with standard chow and water *ad libitum* provided by the animal facilities.

### Design of the Experimental Model of Diabetic Nephropathy and Characterization

Male BTBR ob/ob diabetic mice and their corresponding non-diabetic and non-obese littermates (BTBR WT) were studied over 22 weeks of age (*n* = 6 for each group). These mice rapidly develop morphologic renal lesions characteristic of both early and advanced human DN (Hudkins et al., 2010). Body weight and blood glucose levels were measured by Fisherbrand Precision balance and NovaPro Glu/Ket system (Nova Biomedical, Waltham, MA, USA) after clipping the distal 2–3 mm of the tail-tip. Spot urine samples were collected once a week from all mice and analyzed for albumin and creatinine by the ELISA Kit (cat. nos. ab108792 and ab65340, respectively, Abcam) to obtain the urine albumin/creatinine ratio (ACR). Animals were euthanatized by intraperitoneal anesthetic induction of ketamine (100 mg/kg) and xylazine (10 mg/kg). After anesthetic assessment, the kidneys were perfused *in situ* with saline before removal, and one half of each kidney was fixed in 4% formaldehyde, embedded in paraffin, and used for histological studies, whereas the other half remaining was snap-frozen in liquid nitrogen for renal cortex miRNA studies. Metabolic and renal profile were assessed in serum including: glucose, creatinine, urea, blood urea nitrogen, albumin, triglycerides (TG), total cholesterol (TC), high-density lipoprotein (HDL), and low-density lipoprotein (LDL); aspartate transaminase, alanine aminotransferase, and alkaline phosphatase. The measurement was performed at our institution's central laboratory using Roche Cobas autoanalyzer. Over the disease outcome, glycosuria was measured periodically by test strips (range 50–1,000 mg/dl) and subsequently quantified by urine dilution in institutional autoanalyzer.

### Morphological and Immunohistochemical Studies

A quarter piece of each kidney sample fixed in 4% formaldehyde were embedded in paraffin and cut in serial sections (4–5 μm thickness) for further histological [periodic acid–Schiff (PAS)/Sirius red] and immunohistochemistry (IHC) studies.

### Periodic Acid–Schiff Staining

PAS staining was performed using 0.5% PAS’s Reagent (Sigma-Aldrich) and Hematoxylin (Thermo Scientific) for histopathological assessment in 4 μm sections, as previously described (Varga and Brenner, 2005). The following lesions were evaluated: the degree of mesangial matrix expansion, glomerular hypertrophy (glomerulomegaly), glomerular sclerosis, arteriolar hyalinosis, tubular casts, acute tubular damage, and tubular atrophy, as well as the presence of interstitial inflammatory cells and fibrosis. A semi-quantitative assessment of renal damage at glomerular (glomerulomegaly, mesangial matrix expansion, presence of nodular sclerosis, and arteriolar hyalinosis) and tubulointerstitial (casts, tubular flattening, tubular atrophy, inflammatory infiltrates, and fibrosis) levels as well as the total (sum of both scores) lesions were graded according to their histopathological score (from 0 to 4) as previously described (Zoja et al., 2002). The histological assessment and representative lesions were obtained by epifluorescence optical microscope (Axioscope 5 Workstation compact, Zeiss).

### Sirius Red Staining

Sirius red staining was performed in 5-µm kidney sections using a picrosirius solution [10% of Sirius red (1%) in picric acid], as previously described (Varga and Brenner, 2005). Collagen deposition levels were evaluated by using the Image-Pro Plus software (Bio-Rad) determining the red positive staining per glomerular and interstitial areas in eight randomly chosen fields (×40 and ×20 magnification, respectively) acquired by epifluorescence optical microscope (Axioscope 5Workstation compact, Zeiss).

### Immunohistological Studies

For immunostaining procedure, 4-µm tissue sections were deparaffinized through xylene and hydrated through graded ethanol (100%, 96%, 90%, and 70%) ending in distilled water. Antigens were restored by using PTLink system (DAKO Diagnostic), blocking endogenous peroxidase afterward. Commercial casein solution (DAKO Diagnostic) was used to release non-specific protein bindings (1 h at room temperature), and tissue sections were incubated overnight at 4°C withp-SMAD3 (1/200; ab52903, Abcam) antibody diluted in antibody solution (DAKO Diagnostics). Kidney sections were incubated with the specific HRP secondary anti-body (GENA934, Sigma Chemical) for 1 h followed by Avidin-Biotin Complex incubation (Vector laboratories) for 30 min. To develop signal, samples were incubated with substrate solution and 3,3-diaminobenzidine as a chromogen (Abcam) and counterstained with Carazzi’s hematoxylin (Thermo Fisher Scientific). Specificity was checked by omission of primary antibody (data not shown). Quantification was made by using the Image-Pro Plus software (Bio-Rad) determining the positive relative staining area per total area in five to 10 randomly chosen fields (×40 magnification) acquired by epifluorescence optical microscope (Axioscope 5Workstation compact, Zeiss).

### Total RNA Extraction

Cortex kidney sections were lysed using Tissue Lyser for disaggregation of tissues, and total RNA was obtained using the KingFisher Flex station (Thermo Fisher) and a commercial kit (MagMAX™ mirVana™), following the manufacturer’s specifications; total RNA was quantified using a 2100 Bioanalyzer (Agilent) to test size profiling.

### miRNA Sequencing and Bioinformatic Analysis

For sequencing of small RNAs, 12 libraries (six BTBR WT and six BTBR ob/ob) were prepared according to the instructions of the “NEBNext Multiplex Small RNA Library Prep Set for Illumina” kit (New England Biolabs). The input amount of total RNA to start the protocol was 800 ng of each sample, according to the bioanalyzer measurements in RNA 6000 Nano Chips. The library preparation procedure included a PCR step, which was adjusted to 13 cycles. The libraries obtained were run in 6% PolyAcrylamide Gel, and the region of 135–325 pb (corresponding to small RNA) was selected and assessed using an Agilent 2100 Bioanalyzer with High-Sensitivity DNA chips. The final pool of libraries was denatured prior to being seeded on a flow-cell at a density of 1.38 pM, where clusters were formed and sequenced in a NovaSeq 6000 sequencer using a flow cell SP v1.5 (Illumina). An amount of 18–37 million of single-end reads (mean 24.4 × 10^6^) was obtained per sample. The quality of sequences was assessed using the program FastQC-0.11.7 (http://www.bioinformatics.bbsrc.ac.uk/projects/fastqc). Sequences were then filtered according to size (minimum length of 16) and ambiguities (removing reads with more than 10 Ns within the sequence). To that purpose, the program Prinseq (Schmieder and Edwards, 2011) was used. Next, sequences were mapped against *Mus musculus* genome (release GRCh38) using TopHat (Trapnell et al., 2012; Kim et al., 2013); reads were annotated using the corresponding MmGRCm38.95-gtf file. Numbers of sequences per sample are summarized in the Supplementary Table S1. The whole bioinformatic protocol was executed using the RNAseq pipeline app of the GPRO-suite (Llorens et al., 2011). Total samples (six BTBR WT and six BTBR ob/ob) were used for differential expression analysis, made using Cufflinks/CuffDiff (Trapnell et al., 2012; Kim et al., 2013) to compare the expression of BTBR ob/ob vs. BTBR WT. We filtered out those entries whose detection levels were lower than 40 counts (sum of averages from BTBR W and BTBR ob/ob groups) and finally found a number of 191 microRNA entries which can be considered as positively detected. The bioinformatic study about the possible outcome of miRNA-based regulation was performed using the Ingenuity Pathway Analysis software (IPA, Qiagen).

### RT-qPCR miRNA Validation and mRNA Levels Evaluation

Cortex kidney mRNA from BTBR WT and BTBR ob/ob mice, used to perform the miRNA-seq study, were analyzed by multiple RT-qPCR. For miRNA evaluation, a miRCURY LNA RT kit (Qiagen) was use to obtain cDNA. To confirm miRNA-seq results, qPCR was performed miRCURY LNA miRNA PCR Assays (Qiagen) following the manufacturer’s instructions: miR-802 (YP00205002), miR-34a (YP00204486), and miR-375 (YP00204362); normalized by 5S rRNA (hsa) (YP00203906). For mRNA evaluation, cDNA was obtained by using the reverse transcription kit (Applied Biosystems). Then, a multiplex RT-qPCR was performed using fluorogenic primers design by assay on demand mouse expression products (Applied Biosystems): *Ngal*: Mm01324470_m1, *Mcp1*: Mm00441242_m1, *Acta2*: Mm01546133_m1, and *Tgf-β*: Mm01178820_m1. As endogen control to normalize, *Gapdh:* Mm99999915_g1 was used. miRNA and mRNA copy number were calculated for each sample by the instrument software (ABIPrism 7500 Fast sequence detection PCR system software; Applied Biosystems) using Ct value (“arithmetic fit point analysis for the light cycler”), and the results were expressed in n-fold calculated vs. BTBR WT group.

### Statistical Analysis

Data are expressed as mean ± standard error of the mean (SEM) of each group (*n* = 6 mice/group). Normality distribution was tested by using Shapiro–Wilk test. If the samples followed a Gaussian distribution or not, then means were compared by Student t-test or Mann–Whitney statistical test, respectively. Statistical significance was assumed when a null hypothesis could be rejected at *p* < 0.05. The statistical analysis was performed using the GraphPad Prism software (GrahPad Software).

## Results

### Renal Lesions in the Experimental Model of Advanced Diabetic Nephropathy

**FIGURE 1:**
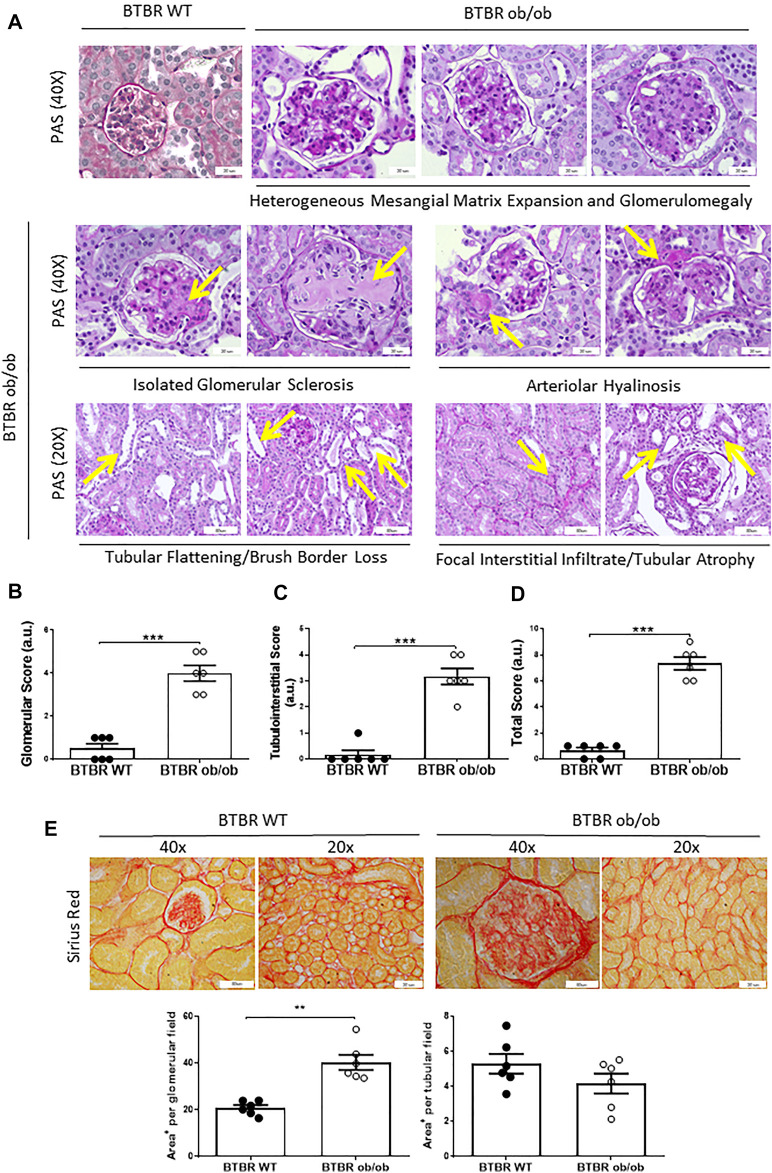
The glomerular and tubulointerstitial features observed in BTBR ob/ob of 22 weeks old and histopathological assessment. **(A)** Representative images of the kidney PAS staining at different magnifications showing the main glomerular (yellow arrows) and tubulointerstitial (green arrows) changes detected. The histopathological assessment was evaluated in glomerular **(B)**, tubulointerstitial **(C)**, and total score **(D)**. **(E)** Representative images of the kidney Sirius red staining at different magnifications (upper) and their quantification (lower). Data are shown as mean ± SEM and graphs bar with scatter dot plots of each group (*n* = 6 mice/group); ***p < 0.001vs. BTBR WT. **p < 0.005 *vs*. BTBR WT. a.u., arbitrary units.

**FIGURE 2:**
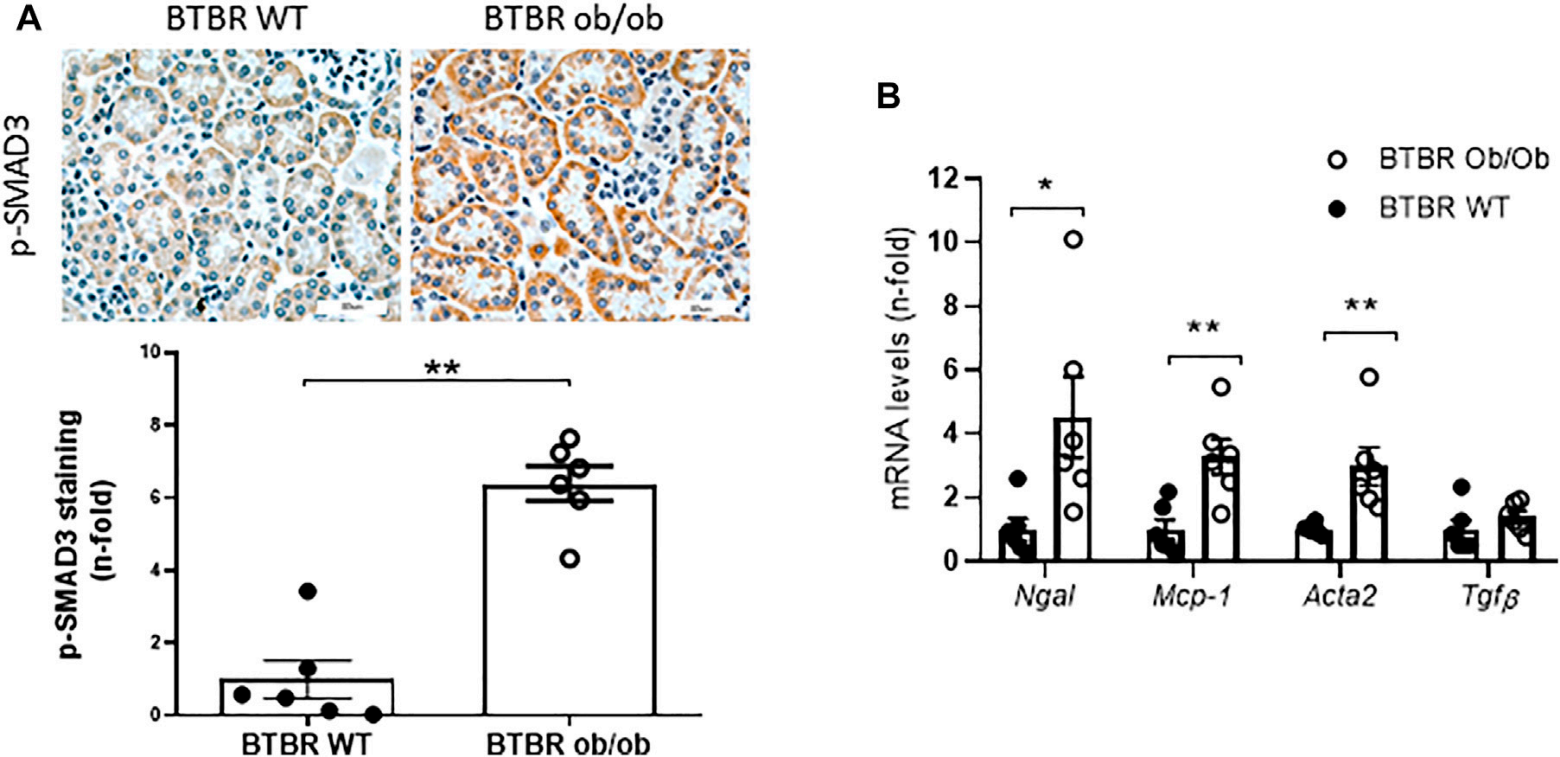
Evaluation of the SMAD pathway activation and inflammatory and fibrotic markers. **(A)** Increased kidney levels of phosphorylated SMAD3 (*p*-SMAD3) were observed in BTBR ob/ob of 22 weeks old compared to BTBR WT. **(B)** Increased levels of *Ngal*, *Mcp-1*, and *Acta2* were observed in the BTBR ob/ob group compared to BTBR WT mice, whereas no differences were detected in *Tgf-β* expression levels. Data are shown as mean ± SEM and graphs bar with scatter dot plots of each group (*n* = 6 mice/group). ***p < 0.001 *vs*. BTBR WT; **p < 0.005 *vs.* BTBR WT; *p < 0.05 *vs.* BTBR WT.

The BTBR ob/ob mice model at 22 weeks of age resembles the kidney damage observed in patients with advanced DN, as previously described (Hudkins et al., 2010; Alpers and Hudkins, 2011). The key features observed after histopathological PAS assessment are shown in the Figure 1A. At the glomerular level, the main hallmarks of kidney damage in diabetic mice are the glomerulomegaly and increased mesangial matrix. Isolated nodular glomerulosclerosis could be detected once as a blue moon in one or two glomeruli throughout the entire renal tissue assessment. Arteriolar hyalinosis was noted by thickening of the arteriolar walls with PAS-positive material at the vascular pole. At the tubulointerstitial level, the presence of cast (isolated PAS-positive), tubular flattening manifested by loss of the tubular cells brush border, focal and/or diffuse inflammatory infiltrate, and tubular atrophy were found. The quantification of kidney damage in BTBR ob/ob mice compared with non-obese non-diabetic BTRB WT mice of the same age used as controls is shown at glomerular (Figure 1B), tubulointerstitial (Figure 1C), and total areas (Figure 1D). According to previous results (Lavoz et al., 2020b), the studied kidneys did not develop tubulointerstitial fibrosis, which was also confirmed by Sirius red staining (Figure 1E). On the other hand, a high activation of the fibrotic-related pathway SMAD, assessed by increased SMAD3 phosphorylated levels (*p*-SMAD3), was found in BTBR ob/ob mice compared to control mice (Figure 2A). At mRNA level, an increased expression of *Acta2*were observed in the BTBR ob/ob mice kidneys compared with control mice, whereas no differences were found in the mRNA levels of *Tgfβ* (Figure 2B). In addition, mRNA levels of several inflammatory-related factors (*Ngal* and *Mcp-1*) were upregulated in the BTBR ob/ob mice kidneys compared to controls (Figure 2B).

To further evaluate changes in renal function, both serum and urinary markers were assessed. As previously described, the BTBR ob/ob mice showed hyperglycemia and increase in body weight at early stages, which were maintained over time (22 weeks) with changes in serum lipids, such as TC, TGs, and HDL in comparison with BTBR WT (Table 1). The BTBR ob/ob mice at 22nd week presented discrete elevation of serum creatinine, severe albuminuria (measured by urinary ACR), and high glycosuria levels as compared to control littermates, revealing kidney dysfunction in diabetic mice (Table 1).

**TABLE 1:** Metabolic and renal parameters in BTBR WT and BTBR ob/ob mice. *p < 0.05 *vs*. BTBR WT; UD, undetectable.

General Parameters	BTBR WT	BTBR ob/ob
Body weight at 22th week (g)	38 ± 0.8	72 ± 1.1*
Glycaemia at 22th week (mg/dl)	151 ± 4	531 ± 24*
Glycosuria (mg/dl)	UD	2190 ± 278
Urinary albumin/creatinine ratio—uACR (mg/g)	50 ± 7.2	850 ± 99*
Serum creatinine (mg/dl)	0.20 ± 0.01	0.27 ± 0.01*
Urea (mg/dl)	43 ± 2.9	42 ± 4.2
Blood urea nitrogen—BUN (mg/dl)	20 ± 1.4	20 ± 1.9
Aspartate transaminase—AST (IU/L)	67 ± 4.8	85 ± 9.2
Alanine transaminase—ALT (IU/L)	12 ± 0.8	44 ± 7.0*
Alkaline phosphatase—AP (IU/L)	34 ± 1.6	89 ± 8.7*
Serum albumin (g/dl)	3.5 ± 0.1	4.3 ± 0.3*
Total cholesterol (mg/dl)	126 ± 4.8	203 ± 19.1*
Triglycerides (mg/dl)	69 ± 8.4	96 ± 11.3*
Low-density lipid—LDL (mg/dl)	6.8 ± 0.8	37 ± 7.4*
High-density lipid—HDL (mg/dl)	107 ± 3.8	147 ± 12.8*

### Renal Cortex of BTBR Ob/Ob Mice Displays a Different miRNA Pattern Expression in Relation to BTBR WT Mice

**FIGURE 3:**
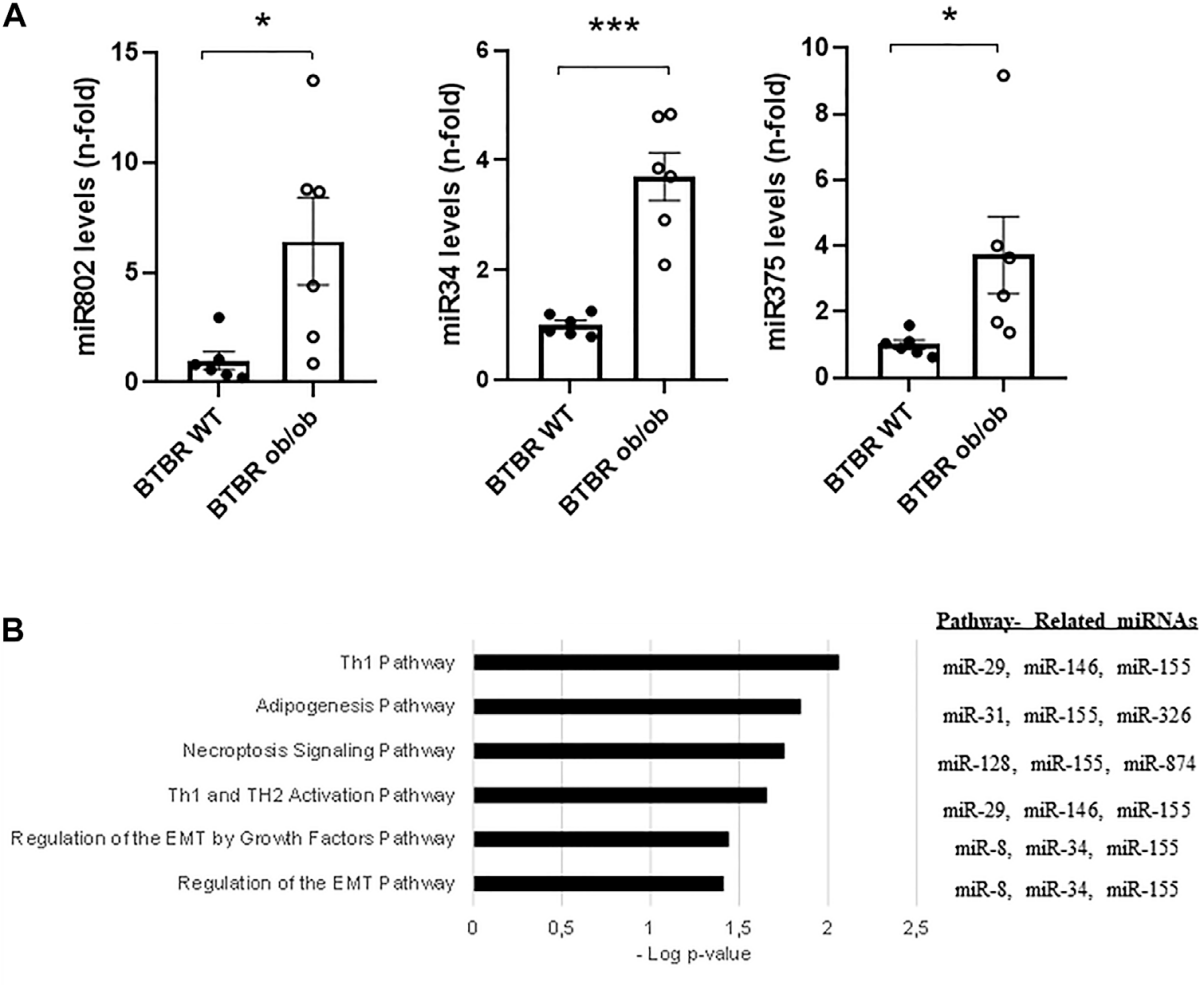
miRNA RT-qPCR validation and assessment of key pathways and miRNAs. **(A)** miRNA relative expression (n-fold vs. BTBR WT) of miR802, miR34a, and miR375. **(B)** Left panel shows a graphic with the top canonical pathways related to the 99 miRNAs that were significantly predicted in the comparative analysis of the cortex kidney of BTBR ob/ob mice vs. BTBR WT, omitting cancer related pathways. Right panel indicates the most related miRNAs to each pathway. Data are shown as mean ± SEM and graphs bar with scatter dot plots of each group (*n* = 6 mice/group). ***p < 0.001vs. BTBR WT; *p < 0.05 *vs*. BTBR WT.

To evaluate the miRNAs potentially involved in the genesis of advanced DN in the BTBR ob/ob mice (22-weeks-old), a miRNA-seq study was performed. The results showed 198 miRNAs whose expression was dysregulated (194 upregulated vs. four downregulated) in the BTBR ob/ob group vs. WT (Supplementary Table S2). Among them, 99 upregulated miRNAs presented q-values < 0.05, whereas none deregulated miRNAs showed significant differences between groups (Table 2). According to the n-fold changes, miR-802 (44.55-fold), miR-34a (13.04-fold), miR-132 (9.68-fold), miR-101a (8.52-fold), and mir-379 (7.89-fold) were some of the most upregulated miRNAs in the BTBR ob/ob group. The elevated expression of several miRNAs (miR-802, miR-34, and miR375) was also confirmed by RT-qPCR (Figure 3A).

**TABLE 2:** List of the 99 miRNAs presenting q-values < 0.05 in the comparative analysis of BTBR ob/ob vs. BTBR WT group.

Ensembl Gene_ID	miRNA	Fold Change	q_value	Ensembl Gene_ID2	miRNA	Fold Change	q_value
ENSMUSG00000076457	miR-802	44.55	0.0180327	ENSMUSG00000077962	miR-874	6.69	0.0336
ENSMUSG00000065493	miR-34a	13.04	0.00063579	ENSMUSG00000076066	miR-223	3.64	0.00431
ENSMUSG00000065537	miR-132	9.68	0.00063579	ENSMUSG00000065610	miR-29a	3.63	0.0281
ENSMUSG00000065451	miR-101a	8.52	0.0467852	ENSMUSG00000077042	miR-574	3.61	0.000636
ENSMUSG00000065498	miR-379	7.89	0.00063579	ENSMUSG00000065611	miR-23a	3.54	0.000636
ENSMUSG00000065507	miR-204	7.84	0.00063579	ENSMUSG00000065439	miR-140	3.54	0.000636
ENSMUSG00000092830	miR-3963	7.56	0.00063579	ENSMUSG00000104618	miR-1839	3.49	0.000636
ENSMUSG00000065477	miR-411	7.24	0.00063579	ENSMUSG00000099036	miR-378c	3.46	0.000636
ENSMUSG00000098973	miR-6236	6.87	0.00063579	ENSMUSG00000076338	miR-181d	3.44	0.000636
ENSMUSG00000065551	miR-210	6.79	0.00063579	ENSMUSG00000065586	miR-96	3.41	0.000636
ENSMUSG00000065616	miR-375	6.18	0.00063579	ENSMUSG00000076255	miR-92b	3.40	0.000636
ENSMUSG00000070102	miR-455	5.93	0.00063579	ENSMUSG00000065418	miR-322	3.36	0.000636
ENSMUSG00000065546	miR-196a-1	5.91	0.00063579	ENSMUSG00000076010	miR-615	3.31	0.000636
ENSMUSG00000065443	miR-196b	5.90	0.00063579	ENSMUSG00000065601	miR-146	3.28	0.0271
ENSMUSG00000065607	miR-331	5.83	0.0293941	ENSMUSG00000065484	miR-130a	3.25	0.000636
ENSMUSG00000065592	miR-145a	5.69	0.00063579	ENSMUSG00000070074	miR-484	3.25	0.0168
ENSMUSG00000076011	miR-652	5.52	0.00063579	ENSMUSG00000065470	miR-149	3.25	0.00325
ENSMUSG00000065402	miR-122	5.37	0.00063579	ENSMUSG00000076062	miR-92–1	3.21	0.0117
ENSMUSG00000076357	miR-653	5.26	0.0266784	ENSMUSG00000065408	miR-31	3.13	0.00569
ENSMUSG00000070076	miR-127	5.18	0.00063579	ENSMUSG00000105972	miR-1843a	3.09	0.00809
ENSMUSG00000092998	miR-5099	5.14	0.00063579	ENSMUSG00000065422	miR-221	3.06	0.000636
ENSMUSG00000065417	miR-340	5.06	0.00063579	ENSMUSG00000065397	miR-155	3.06	0.000636
ENSMUSG00000070139	miR-532	5.05	0.0237531	ENSMUSG00000065580	miR-15b	3.03	0.00364
ENSMUSG00000106465	miR-374c	5.01	0.00114916	ENSMUSG00000065510	miR-361	3.03	0.000636
ENSMUSG00000065556	miR-101b	5.00	0.00063579	ENSMUSG00000065471	miR-222	3.00	0.000636
ENSMUSG00000065500	miR-10b	4.84	0.00364284	ENSMUSG00000105497	miR-191	3.00	0.00204
ENSMUSG00000105200	miR-378a	4.68	0.00431115	ENSMUSG00000065528	miR-320	3.00	0.000636
ENSMUSG00000065431	miR-186	4.52	0.00063579	ENSMUSG00000065464	miR-185	2.94	0.00325
ENSMUSG00000076049	miR-598	4.50	0.0420373	ENSMUSG00000065587	miR-34c	2.92	0.000636
ENSMUSG00000065593	miR-339	4.44	0.00063579	ENSMUSG00000076361	miR-182	2.89	0.000636
ENSMUSG00000065503	miR-351	4.43	0.00063579	ENSMUSG00000065462	miR-200c	2.86	0.0196
ENSMUSG00000065446	miR-139	4.39	0.00063579	ENSMUSG00000076122	miR-503	2.83	0.000636
ENSMUSG00000065520	miR-128-1	4.37	0.00697744	ENSMUSG00000070130	miR-328	2.83	0.000636
ENSMUSG00000105458	miR-3074-2	4.23	0.00063579	ENSMUSG00000065542	miR-224	2.80	0.00502
ENSMUSG00000065396	miR-99b	4.22	0.0175788	ENSMUSG00000098756	miR-378d	2.71	0.000636
ENSMUSG00000065476	miR-30b	4.01	0.00114916	ENSMUSG00000065619	miR-183	2.68	0.000636
ENSMUSG00000065489	miR-365-2	4.01	0.00063579	ENSMUSG00000076460	miR-744	2.64	0.016
ENSMUSG00000065429	miR-345	3.99	0.00063579	ENSMUSG00000098343	miR-6240	2.63	0.000636
ENSMUSG00000076398	miR-676	3.95	0.00063579	ENSMUSG00000065411	miR-195a	2.56	0.000636
ENSMUSG00000065612	miR-151	3.91	0.00063579	ENSMUSG00000065582	miR-194–2	2.49	0.00536
ENSMUSG00000099169	miR-7j	3.90	0.00063579	ENSMUSG00000065518	miR-423	2.37	0.00974
ENSMUSG00000065543	miR-330	3.89	0.00063579	ENSMUSG00000065574	miR-203	2.26	0.01
ENSMUSG00000065548	miR-29c	3.84	0.0161905	ENSMUSG00000070106	miR-363	2.13	0.00431
ENSMUSG00000065515	miR-152	3.77	0.00063579	ENSMUSG00000093011	miR-100	2.07	0.0476
ENSMUSG00000080645	miR-1198	3.77	0.00063579	ENSMUSG00000105220	miR-497	2.0	0.00754
ENSMUSG00000076376	miR-674	3.77	0.00063579	ENSMUSG00000065495	miR-150	1.93	0.00864
ENSMUSG00000076140	miR-542	3.76	0.00063579	ENSMUSG00000065613	miR-92–2	1.85	0.0168
ENSMUSG00000105196	miR-142	3.75	0.00063579	ENSMUSG00000065395	miR-193a	1.80	0.0271
ENSMUSG00000065571	miR-326	3.71	0.0325777	ENSMUSG00000065532	miR-187	1.73	0.0301
ENSMUSG00000065560	miR-148b	3.71	0.00063579	—	—	—	—

### Functional Analysis of the miRNA-Seq Results

**FIGURE 4:**
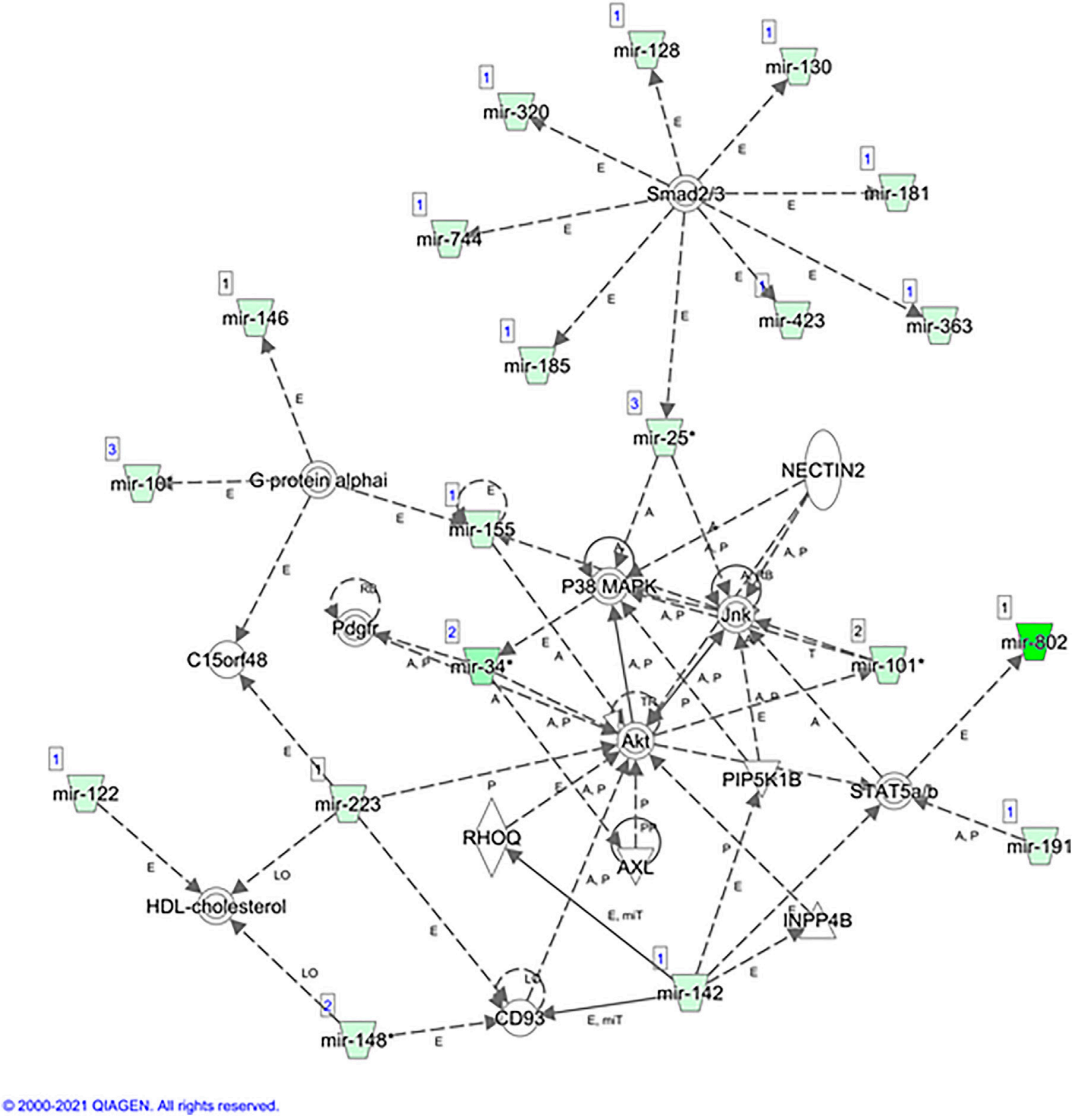
Predicted network. Interaction network of miRNAs and genes related to connective tissue disorders, gene expression, and inflammatory diseases, obtained with the IPA bioinformatic analysis for the 99 miRNAs significantly upregulated in the BTBR ob/ob mice group.

A subsequent Ingenuity Pathway Analysis (IPA) performed with the IPA software and using the 99 miRNAs (q-values < 0.05) displayed a set of canonical pathways related to these miRNAs (Supplementary Table S3). Although cancer-related pathways were the most highlighted, we decided to focus on other potential biological processes that could be deregulated in our study. This new analysis identified T helper (Th) immune responses (Th1 and Th2), adipogenesis, necroptosis, and EMT pathways as the top canonical pathways related to these miRNAs (Figure 3B). The main predicted network obtained was related to connective tissue disorders, gene expression, and inflammatory diseases (Figure 4). The top upstream regulator analysis showed the miRNA biogenesis components AGO2 and DICER1 as the most predicted factors to be regulated by the studied miRNAs, with HNF4a, SMAD2/3, and TNFRSF1B being the next factors of the list (Table 3).

**TABLE 3:** Top upstream regulator predicted molecules for the 99 miRNAs significantly upregulated in the kidney cortex of BTBR mice.

Upstream regulator	Molecule type	*p*-value of overlap	Target molecules in dataset
AGO2	Translation regulator	6.88E-68	mir-10, mir-101, mir-122, mir-128, mir-130, mir-139, mir-148, mir-15, mir-181, mir-182, mir-185, mir-186, mir-187, mir-188, mir-193, mir-194, mir-203, mir-204, mir-221, mir-23, mir-25, mir-28, mir-29, mir-320, mir-328, mir-330, mir-339, mir-34, mir-340, mir-361, mir-365, mir-379, mir-423, mir-484, mir-598, mir-652, mir-676, mir-8, mir-802
DICER1	Enzyme	9.67E-39	mir-10, mir-101, mir-122, mir-132, mir-139, mir-142, mir-145, mir-146, mir-148, mir-15, mir-150, mir-155, mir-181, mir-182, mir-188, mir-193, mir-194, mir-196, mir-204, mir-23, mir-25, mir-29, mir-30, mir-331, mir-34, mir-361, mir-365, mir-378, mir-497, mir-8, Mir7j
HNF4A	Transcription regulator	9.48E-20	mir-10, mir-101, mir-130, mir-140, mir-142, mir-148, mir-15, mir-181, mir-193, mir-194, mir-203, mir-28, mir-31, mir-34, mir-365, mir-455, mir-484, mir-497, mir-574, mir-8, mir-802
Smad2/3	Group	0.0000000000000039	mir-128, mir-130, mir-181, mir-185, mir-25, mir-320, mir-363, mir-423, mir-744
TNFRSF1B	Transmembrane receptor	0.000000000000014	mir-10, mir-101, mir-130, mir-221, mir-28, mir-30, mir-320, mir-322, mir-326, mir-34, mir-96
NF2	Other	0.00000000000225	mir-10, mir-101, mir-15, mir-188, mir-28, mir-29
MTDH	Transcription regulator	0.0000000036	mir-101, mir-15, mir-182, mir-28, mir-340
E2F3	Transcription regulator	0.00000000873	mir-10, mir-145, mir-15, mir-221, mir-23, mir-25
KHDRBS1	Transcription regulator	0.0000000379	mir-101, mir-142, mir-29, mir-339
PPARA	Ligand-dependent nuclear receptor	0.0000000764	mir-10, mir-101, mir-130, mir-146, mir-148, mir-182, mir-203, mir-25, mir-30, mir-34, mir-378, mir-8
SPI1	Transcription regulator	0.00000129	mir-142, mir-223, mir-322, mir-351, mir-503, mir-542, mir-8
ETS2	Transcription regulator	0.00000173	mir-142, mir-155, mir-223, mir-29
PIK3CA	Kinase	0.00000258	mir-210, mir-221, mir-34
HMGA1	Transcription regulator	0.00000426	mir-101, mir-196, mir-29, mir-331
Gulo	Enzyme	0.000012	mir-15, mir-30, mir-322
SMARCA4	Transcription regulator	0.0000181	mir-145, mir-28, mir-30, mir-34, mir-674
E2F1	Transcription regulator	0.0000241	mir-10, mir-145, mir-15, mir-221, mir-23, mir-25
IGF1R	Transmembrane receptor	0.0000451	mir-10, mir-127, mir-132, mir-139, mir-196, mir-34, mir-379
REST	Transcription regulator	0.0000607	mir-139, mir-148, mir-203, mir-204
MYC	Transcription regulator	0.000124	mir-145, mir-188, mir-29, mir-339, mir-34, mir-365, mir-378
LTB4R	G protein–coupled receptor	0.00016	mir-10, mir-146, mir-155
AGO1	Translation regulator	0.000178	mir-203, mir-8
SLC9A3R1	Other	0.000226	mir-145, mir-155, mir-221
PKD1	Ion channel	0.000236	mir-10, mir-182, mir-204, mir-30, mir-8, mir-96
TNFSF12	Cytokine	0.000327	mir-146, mir-23, mir-322, mir-455
INSR	Kinase	0.000835	mir-10, mir-127, mir-132, mir-139, mir-196, mir-34, mir-379

## Discussion

In the present study, miRNA-seq analysis performed in the renal cortex of an advanced model of DN in 22-week-old BTBR ob/ob mice pointed out a set of 99 miRNA significantly increased compared to non-diabetic non-obese controls of the same age. These miRNAs could potentially be involved in the genesis and progression of DN and, therefore, used as potential biomarkers and/or therapeutic targets in this clinical condition. Our results disclosed increased levels not only of several miRNAs previously described in diabetic complications but also of other miRNAs related to different pathological processes. Interestingly, the functional predictive analysis highlighted inflammatory and immune processes as one of the most relevant pathways related to these miRNAs, remarking the importance of inflammation in the pathogenesis of kidney damage associated to diabetes.

Among the most deregulated miRNAs, miR-802 was, by far, the most upregulated in BTBR ob/ob mice. miR-802 is located on the 21st chromosome, and it has widely been associated with cancer in recent years, although showing opposite effects depending on the organ involved (Zhang et al., 2017; Feng et al., 2020; Ni et al., 2020; Wang et al., 2020; Wu et al., 2020). Interestingly, high levels of miR-802 have also been described in diabetic patients and in obesity-induced experimental nephropathy (Sun et al., 2019; Zhang et al., 2020). In addition, miR-101 and miR-375, both elevated in our miRNA-seq study, have also been involved in T1D and T2Donset and progression (Patoulias, 2018; Santos et al., 2019). Thus, increased levels of circulating miR-802, miR-101, and miR-375 have been proposed as potential biomarkers for T2D in humans (Higuchi et al., 2015). Our results point out the potential direct participation of these miRNAs in the DN progression. Mechanistically, an increase in the hepatic oxidative stress induced by miR-802 was proposed as a potential mechanism implicated in the insulin resistance observed in high-fat diet (HFD)–fed mice (Yang et al., 2019). Regarding the kidney, a recent study demonstrated that miR-802 causes nephropathy in HFD mice by suppressing NF-κB–repressing factor (Sun et al., 2019). Other authors demonstrated that miR-802 overexpression in two obese murine models, HFD and Lepr (db/db) mice, impairs glucose metabolism by silencing Hnf1b (Kornfeld et al., 2013). Our study provides further support for the direct involvement of mir-802 in DN and extends these findings to another experimental model that closely replicates the key histopathological features observed in human DN.

Our results additionally highlighted another set of miRNAs previously described to participate in the pathogenesis of DN. Thus, several authors suggested miR-200b/c and mir-29c as key therapeutic targets in experimental DN in db/db mice (Long et al., 2011; Park et al., 2013). Accordingly, we observed a significant increase in miR-200c and miR-29c levels in BTBR ob/ob mice. On the contrary, although previous studies demonstrated that miR-146 deletion accelerate DN progression in a murine streptozotocin (STZ)–induced diabetes model (Bhatt et al., 2016b; Sankrityayan et al., 2019), we found out an increase in miR-146 levels in the cortex kidney of BTBR ob/ob mice. This disparity could be explained by the differences between the experimental diabetes model used, one induced by STZ resembling T1D and the other by DN generated in BTBR ob/ob mice that resembles advanced human DN lesions in a T2D and metabolic disorder milieu (King, 2012). Increased miR-132 circulating levels have been associated with T2D and nephropathy and approaches to targeting it did impair blood glucose and improved insulin secretion (Bijkerk et al., 2015; Bijkerk et al., 2019; Florijn et al., 2019). Accordingly, we found out increased miR-132 expression in the kidney of BTBR ob/ob mice. In a recent study, a protective role of miR-379 deletion was described in STZ-induced DN in mice (Kato et al., 2021), with this miRNA being one of the most upregulated in our study. Another example of diabetes-related miRNAs in the present report is miR-204, whose genetic deletion improved glycemic control despite obesity in db/db mice (Gaddam et al., 2020). On the other hand, some miRNAs described to play a protective role in DN progression are miR-29b (Chen et al., 2014), miR-34c (Liu et al., 2015), and miR-26a (Koga et al., 2015). In the present study we did not observe differences in miR-29b andmiR-26a levels, whereas miR-34c was increased in the BTBR ob/ob. Whereas the mentioned miR-34c (Liu et al., 2015) protective effects were described in cultured podocytes, our study was performed in the renal cortex of diabetic mice, which could explain the apparent discrepancies among both studies.

Besides the potential individual effects exerted by the above-described miRNAs, our bioinformatic analysis pointed out Th1 and Th2 responses as the most common associated pathways to our miRNA-list, immediately after cancer related pathways that were not considered here as commented in the results section. The intensive research in the immunology field during the last years have markedly contributed to unravel the role of immune cells in many diseases, remarking the important role of Th subtypes, including Th1, Th2, Th17, and T regulatory (Treg) in many human diseases (Raphael et al., 2015). Importantly, Th subtype differentiation is a tightly regulated process, and mixed phenotypes can be found depending on the pathological conditions and can be modified by therapeutic interventions (DuPage and Bluestone, 2016; Gagliani and Huber, 2017). In this sense, Th1 and Th17 cells are characteristic of proinflammatory conditions, as described in immune mediated-disorders and chronic inflammatory diseases, whereas Treg cells exert protective anti-inflammatory actions (Harrington et al., 2005; Saigusa et al., 2020). Many experimental studies targeting Th17 immune response by different approaches, including neutralizing antibodies against the effector cytokine IL-17A or its soluble receptor, pharmacological inhibitors of RORγt, the main transcription factor driver of Th17 differentiation or drugs modulating Th17/Treg balance, like vitamin D agonist, as well as studies using genetically modified mice, have remarked the importance of Th17/IL-17A in the pathogenesis of chronic inflammatory diseases, including immune and non-immune renal damage (Orejudo et al., 2019; Lavoz et al., 2020a; Marchant et al., 2020; Rodrigues-Diez et al., 2021). Among the wide range of pathways implicated in DN generation and progression, we recently proposed that Th17 immune response could be playing a main role in DN in humans and BTBR ob/ob diabetic model (Lavoz et al., 2020a). In addition, we have previously demonstrated that administration of an IL-17A neutralizing antibody in BTBR ob/ob mice, starting when renal dysfunction and structural alterations were already present, caused a beneficial effect, restoring renal damage parameters, mainly due to inhibition of NF-κB/inflammation in the diabetic kidney (Lavoz et al., 2019). Other studies support the beneficial effects of IL-17A reduction in experimental DN in other mice models, as STZ-induced diabetes and autoimmune diabetes in NOD mice (Emamaullee et al., 2009; Kuriya et al., 2013; Kim et al., 2015; Tong et al., 2015).

Regarding the role of miRNAs in the regulation of the Th17 response, several authors described their participation in different pathologies including autoimmune diseases. In this sense, miR-20b and miR-30a suppress Th17 differentiation in experimental autoimmune diseases by targeting RORγt and STAT3 and IL-21R, respectively (Zhu et al., 2014; Qu et al., 2016). Similarly, in another autoimmune disease, miR-155-3p targets two genes (Dnaja2 and Dnajb1) that negatively regulated Th17 differentiation contributing to potentiate the Th17 response (Mycko et al., 2015). miR-146a elicits a different modulation of the Th17 response depending on the context. In experimental autoimmune encephalomyelitis, miR-146a reduced Th17 differentiation by targeting IL-6 and IL-2 (Li B. et al., 2017). However, a more recent study suggested that miR-146a-5p promotes Th17 cell differentiation by targeting the metalloprotease ADAM17 in the primary Sjögren’s syndrome (Wang X. et al., 2021). Increased levels of mir-34a, the second miRNA mostly upregulated in our miRNA-seq, have been described as an inductor of Th17 response by targeting FOXP3 in rheumatoid arthritis and systemic lupus erythematosus patients (Xie et al., 2019). Moreover, miR-34a may induce increased level of IL-17 and other proinflammatory cytokines *via* SIRT1 direct targeting and subsequent induction of NF-κB and the downstream pathway (Karbasforooshan and Karimi, 2018). Remarkably, miR-802 increases Th17 immune response by targeting the suppressor of cytokine signaling (SOCS5) in inflammatory bowel disease (Yao et al., 2020). Apart from immune diseases, the relation between miRNAs and Th17 has been demonstrated in other pathologies. In hepatocellular carcinoma–derived Th17 cells, a recent article showed increased miR-132 levels, another upregulated miRNA in our study, and demonstrated that the use of a miR-132 mimic accelerates Th17 differentiation *in vitro* (Feng et al., 2021). At the kidney level, there are few studies on the role of miRNAs in Th17 differentiation (Lavoz et al., 2020a). In this sense, in experimental crescentic glomerulonephritis, miR-155 participated in Th17 immune response and tissue injury (Krebs et al., 2013), and their deficiency attenuated the renal damage in hyperglycemia-induced nephropathy by promoting nephrin acetylation (Lin et al., 2015).

Interestingly, some of the miRNAs related to Th cell responses also participate in the diabetic pathology and were modified in our miRNA-seq study. Thereby, as mentioned above, miR-146a was proposed to be related to T2D susceptibility after a meta-analysis performed in a total of 12 studies that reveal a downregulation of miR-146a circulating levels in diabetic subjects in comparison to normal ones (Alipoor et al., 2017). In a similar manner, miR-155 was found downregulated in PBMC obtained from type 2 diabetic patients (Corral-Fernández et al., 2013). However, at the kidney level, both miR-155 and miR-146a were increased more than five-fold in DN patients compared with the controls as well as in experimental type 2 DN rat models (Huang et al., 2014). In the present study, both miR-155 and miR-146 were increased in the cortex kidney of BTBR ob/ob mice and were also related to the Th1 and Th2 responses. All the DN-related miRNAs that increased as found in our experimental study, combined with the fact that some of them have also been described to participate in the inflammatory/immune responses, including Th17, support our previous findings on the role of Th17 in DN (Lavoz et al., 2019) and pave the way to future studies modulating miRNAs in this clinical condition.

Other canonical pathway highlighted in our miRNA-seq studies was adipogenesis. Although there are a number of publications relating miRNAs in adipose tissue (Heyn et al., 2020), limited articles relating miRNA and adipogenesis in kidney tissue have been published so far. Recently, we demonstrated the expression of fatty acid influx/efflux markers and the presence of lipid droplets expressing perilipin-1 at both glomerular and tubulointerstitial levels in the BTBR ob/ob model (Opazo-Ríos et al., 2020b). Therefore, the present results, showing miRNAs expression related to adipocyte differentiation and lipid storage in renal tissue, constitute a novel hallmark and opens new ways to investigate the progression of type 2 DN in future studies.

Necroptosis, a form of cell death (Linkermann and Green, 2014; Newton and Manning, 2016), was another pathway remarked in our miRNA-seq studies. Necroptosis is a type of programmed necrosis characterized by the activation of receptor-interacting protein (RIP) 1 and 3 and by damage-associated molecular pattern–induced inflammation (Linkermann and Green, 2014; Newton and Manning, 2016). In kidney diseases, necroptosis has been proposed as a key mechanism involved in cell death in acute kidney damage (AKI), as described in the experimental models of renal ischemia/reperfusion injury, folic acid-induced AKI, and cisplatin nephropathy (Linkermann et al., 2013; Linkermann and Green, 2014; Xu et al., 2015; Martin-Sanchez et al., 2017; Martin-Sanchez et al., 2018). However, data about necroptosis in diabetes and experimental DN, in particular, are very limited. In this sense, in cultured podocytes, high-glucose concentrations elicited necroptosis (Xu et al., 2019). The inhibitor of necroptosis necrostatin-1 (nec-1) reduced visceral fat deposition and restored cognitive function and brain damage, but it did not result in any improvement on insulin sensitivity in prediabetic HFD rats (Jinawong et al., 2020). RIPK3 deficiency alleviated myocardial injury, improved cardiac function, and attenuated necroptosis in mice with STZ-induced diabetic cardiomyopathy (Chen et al., 2021). Our results suggest that necroptosis could be another pathway involved in kidney damage in diabetes to be explored.

In response to an insult, tubular epithelial cells undergo several changes, including loss of cell-to-cell contact and the polarized epithelial phenotype, leading to tubular dysfunction (Ruiz-Ortega et al., 2020). These changes in tubular cell phenotype are described as partial EMT and can contribute to renal damage progression, including in DN (Ruiz-Ortega et al., 2020). Recent studies have pointed out the importance of tubular damage, besides glomerular damage, in the genesis and progression of DN (Rayego-Mateos et al., 2021). In this sense, SGLT2i, besides acting as antihyperglycemic drugs, exert kidney protective effects in type 2 diabetic patients by acting in the proximal tubule reducing sodium reabsorption, probably by improving mitochondrial function (Shirakawa and Sano, 2020) and restoring tubular cell phenotype (Li J. et al., 2020; Das et al., 2020). Many data suggest the involvement of miRNAs in phenotype changes associated to diabetes and fibrotic conditions (Giordo et al., 2021). Our miRNA-seq data, showing that EMT related-processes are also connected to the upregulated miRNAs in advanced experimental DN, support the importance of tubular cells in the progression of kidney damage in DN and suggest that strategies targeting tubular damage should be investigated in DN.

Our bioinformatic analysis has also revealed that the most predictive factors to be regulated by the studied miRNAs are AGO2 and DICER1, both involved in miRNAs regulation, with other targets HNF4α, SMAD2/3 (a key pathway in fibrotic processes) (Tuleta and Frangogiannis, 2021), and TNFRSF1B being a member of the TNF Receptor (TNFR) Superfamily, also known as TNFR2 (So and Ishii, 2019). HNF4α dysfunction has been associated with metabolic disorders including diabetes (Niehof and Borlak, 2008; David-Silva et al., 2013). Mutations in HNF4α and HNF1α cause an autosomal dominant form of DM and tubular dysfunction (Terryn et al., 2016). Developmental studies have unveiled that HNF4α regulates the expression of key genes involved in differentiation and reabsorption in proximal tubules (Marable et al., 2020). TNFRSF1B binds to TNF-α and plays a critical role in immune regulation (So and Ishii, 2019) and kidney damage (Speeckaert et al., 2012). Remarkably, circulating TNFR2 levels are robust predictors of early and late renal function decline leading to ESRD in type 1 and type 2 DN patients (Carlsson et al., 2016; Niewczas et al., 2019).

To sum up, our results unveil a battery of miRNAs controlling key genes involved in adipogenesis, inflammation, immune response, necroptosis, and EMT, which constitute key mechanisms involved in the genesis and progression of DN. These data could potentially be relevant for the design of therapeutic approaches to this dreadful disease.

## Data Availability

The datasets generated for this study can be found in the NCBI SRA archive with BioProject record PRJNA759746 (https://www.ncbi.nlm.nih.gov/bioproject/PRJNA759746/) and BioSample records SRX11993569, SRX11993570, SRX11993571, SRX11993572, SRX11993573, SRX11993574, SRX11993575, SRX11993576, SRX11993577, SRX11993578, SRX11993579, and SRX11993580.
