# Supplementary material for: Kidney microRNA Expression Pattern in Type 2 Diabetic Nephropathy in BTBR Ob/Ob Mice
Source: Front Pharmacol. 2022 Mar 16;13:778776. doi: 10.3389/fphar.2022.778776 (PMC8966705; doi:10.3389/fphar.2022.778776)
Supplement: Supplementary file 2 [file Table1.DOCX]

**Supplementary material**

**Supplementary Table 1**: Total number of reads mapping against the 199 miRNAs found as positively expressed (data per sample). P value = 0.17

| **Columna1** | **BTBR WT 1** | **BTBR WT 2** | **BTBR WT 3** | **BTBR WT 4** | **BTBR WT 5** | **BTBR WT 6** | **Total** | **SD** |
| --- | --- | --- | --- | --- | --- | --- | --- | --- |
| Total reads | 638950 | 236498 | 423062 | 285083 | 296639 | 308275 | 2188507 | 147790 |
| **Columna1** | **BTBR ob/ob 1** | **BTBR ob/ob 2** | **BTBR ob/ob 3** | **BTBR ob/ob 4** | **BTBR ob/ob 5** | **BTBR ob/ob 6** | **Total** | **SD** |
| Total reads | 844676 | 956537 | 851414 | 263621 | 193884 | 409042 | 3519173 | 335753 |
