# Supplementary material for: Kidney microRNA Expression Pattern in Type 2 Diabetic Nephropathy in BTBR Ob/Ob Mice
Source: Front Pharmacol. 2022 Mar 16;13:778776. doi: 10.3389/fphar.2022.778776 (PMC8966705; doi:10.3389/fphar.2022.778776)
Supplement: Supplementary file 3 [file Table3.DOCX]

**Supplementary material**

**Supplementary table 3**: Top canonical pathways related to the 99 miRNAs significantly upregulated in the comparative analysis of the cortex kidney of BTBR ob/ob mice *vs*. BTBR WT.

| **Canonical Pathway** | **p-value** |
| --- | --- |
| Cancer Drug Resistance By Drug Efflux | 2.93E-06 |
| HOTAIR Regulatory Pathway | 3.20E-03 |
| Th1 Pathway | 8.66E-03 |
| Adipogenesis Pathway | 1.42E-02 |
| Necroptosis Signaling Pathway | 1.76E-02 |
| Th1 and TH2 Activation Pathway | 2.21E-02 |
| Regulation of the EMT by Growth Factors Pathway | 3.62E-02 |
| Regulation of the EMT Pathway | 3.87E-02 |
